# Charcot-Marie-Tooth disease: from historical landmarks in Brazil to current care perspectives

**DOI:** 10.1055/s-0043-1770348

**Published:** 2023-08-23

**Authors:** Eduardo Boiteux Uchôa Cavalcanti, Rita de Cássia Carvalho Leal, Wilson Marques Junior, Osvaldo José Moreira do Nascimento

**Affiliations:** 1 Rede SARAH de Hospitais de Reabilitação, Unidade Lago Norte, Ambulatório de Doenças Neuromusculares, Brasília DF, Brazil.; 2 Berenstein Medicina Diagnóstica, Recife PE, Brazil.; 3 Universidade de São Paulo, Faculdade de Medicina de Ribeirão Preto, Departamento de Neurologia, Ribeirão Preto SP, Brazil.; 4 Universidade Federal Fluminense, Faculdade de Medicina, Serviço de Neurologia, Niterói RJ, Brazil.

**Keywords:** Charcot-Marie-Tooth disease, Hereditary Sensory and Motor Neuropathy, Peripheral Nervous System diseases, Doença de Charcot-Marie-Tooth, Neuropatia Hereditária Motora e Sensorial, Doenças do Sistema Nervoso Periférico

## Abstract

Hereditary motor and sensory neuropathy, also known as Charcot-Marie-Tooth disease (CMT), traditionally refers to a group of genetic disorders in which neuropathy is the main or sole feature. Its prevalence varies according to different populations studied, with an estimate between 1:2,500 to 1:10,000. Since the identification of *PMP22* gene duplication on chromosome 17 by Vance et al., in 1989, more than 100 genes have been related to this group of disorders, and we have seen advances in the care of patients, with identification of associated conditions and better supportive treatments, including clinical and surgical interventions. Also, with discoveries in the field of genetics, including RNA interference and gene editing techniques, new treatment perspectives begin to emerge. In the present work, we report the most import landmarks regarding CMT research in Brazil and provide a comprehensive review on topics such as frequency of different genes associated with CMT in our population, prevalence of pain, impact on pregnancy, respiratory features, and development of new therapies.

## Resumo

A neuropatia sensitivo-motora hereditária, também conhecida como doença de Charcot-Marie-Tooth (CMT), tradicionalmente se refere a um grupo de doenças genéticas em que a neuropatia é a principal ou única manifestação. Sua prevalência varia de acordo com as diferentes populações estudadas, com estimativa entre 1:2.500 a 1:10.000. Desde a identificação da duplicação do gene *PMP22* no cromossomo 17, por Vance et al., em 1989, mais de 100 genes foram relacionados a esse grupo de doenças e temos visto avanços no atendimento aos pacientes, com identificação de condições associadas e melhores tratamentos de suporte, incluindo intervenções clínicas e cirúrgicas. Além disso, com as descobertas no campo da genética, incluindo técnicas de interferência de RNA e de edição genética, novas perspectivas de tratamento começaram a surgir. No presente trabalho, relatamos os marcos mais importantes sobre a pesquisa de CMT no Brasil e fornecemos uma revisão abrangente sobre tópicos como frequência de diferentes genes associados à CMT em nossa população, prevalência de dor, impacto na gravidez, alterações respiratórias e desenvolvimento de novas terapias.

## INTRODUCTION

Hereditary motor and sensory neuropathy, also known as Charcot-Marie-Tooth disease (CMT), traditionally refers to a group of genetic disorders in which neuropathy is the main or sole feature. 1 2 According to previously studies, the estimated prevalence of CMT ranges from 1:2,500 to 1:10,000 and its phenotypic presentation is characterized by slowly progressive motor and sensory symptoms, ranging from early onset severe forms to milder cases with onset in adulthood. 3 4 5

Since the identification of *PMP22* gene duplication on chromosome 17 by Vance et al., 6 in 1989, more than 100 genes have been related to this group of disorders. 7 8 9 10 Advances in the identification of disease-causing variants in several new genes not previously related to CMT, some of them being allelic to other neurological diseases like hereditary ataxias, hereditary spastic paraparesis, and motor neuronopathies, and reports of disease-causing variants in CMT-related genes in individuals with complex neurological phenotypes, demonstrates that there is an increasing overlap between CMT and other groups of neurogenetic disorders, with some patients initially presenting with an isolated neuropathy, but evolving to a more complex phenotype over time. 1 2 11 12

Another crucial point is that despite the use of innovative technologies, a significant percentage of patients with CMT and other hereditary neuropathies remains without a confirmatory molecular diagnosis. 9 12 13 14 This fact corroborates the limitation of current techniques for the diagnosis of CMT and raises questions about the possibility of variants in non-coding regions of the genome, repeat expansions mutations, and polygenic inheritance patterns. 14

In recent decades, we have also seen advances in the care of patients with CMT, with identification of associated conditions and better supportive treatments, including clinical and surgical interventions. In addition, with discoveries in the field of genetics, including RNA interference and gene editing techniques, new treatment perspectives begin to emerge. 15 16

We report the most import landmarks about CMT research in Brazil and provide a comprehensive review on topics such as frequency of different genes associated with CMT in our population, prevalence of pain, issues on pregnancy, respiratory features, and development of new therapies.

## METHODS

A search was conducted in the following databases for studies published in Portuguese and English from 2012 to 2022: PubMed, ScienceDirect, and Scientific Electronic Library Online (SciELO). The search process included articles that described the association between *hereditary motor and sensory neuropathy* OR *Charcot-Marie-Tooth disease* AND *epidemiology* , *gene frequency* , *pain* , *pregnancy* , *preimplantation* *genetic* *diagnosis* , *respiratory* *function* and *treatment* . Articles and guidelines describing the main points regarding the care, the use of innovative technologies, and the history of CMT in Brazil were also included. In total, 80 articles were selected.

### Landmarks in Brazil

Professor Paulino Longo, from Escola Paulista de Medicina (São Paulo, SP), was the first to call attention to this group of disorders in Brazil, in 1943, when he and his assistants, Paulo Pinto Pupo and Dante Giorgi, described three related individuals with CMT phenotype. At the time, they referred to this condition as ‘Charcot-Marie amyotrophy’. An interesting fact is that two of the three brothers had increased reflexes and one of them also had the Babinski sign bilaterally. 17 In 1944, Professor Bernardo Couto, from the Instituto de Neurologia Deolindo Couto, Universidade Federal do Rio de Janeiro (Rio de Janeiro, RJ), presented two cases of CMT, preferring to call it “Charcot-Marie neuromedullary amyotrophy” 18 .

Later, in 1962, Professor José Antonio Levy, from Faculdade de Medicina of the Universidade de São Paulo (São Paulo, SP) reported four cases of CMT evaluated at the institution. It is noteworthy that after considering the anatomopathological pattern, the author concluded that this disease could be considered a neuromyopathy, as some muscle histopathological changes were considered primary, while others were secondary to peripheral motor neuron injury. 19

In the decades that followed, case reports were published by several Brazilian researchers, including the association of CMT with Klippel-Feil syndrome, arthrogryposis multiplex, optic atrophy, and glaucoma. 20 21 22 23

In 1995, Professors Marcos de Freitas and Osvaldo J. M. Nascimento, from Universidade Federal Fluminense (Niterói, RJ), published two articles, the first describing the findings of neurophysiological studies performed in 45 individuals with CMT and the second with the characteristics found in the sural nerve biopsy of 41 patients. The authors did not find a relationship between the intensity of the clinical picture and the loss of myelin fibers in CMT1, but there was association between decrease in motor nerve conduction velocities and in the number of nerve fibers. In CMT2, they did not observe a relationship between the clinical picture, motor nerve conduction velocities, and nerve biopsy findings. 21 24

### Frequency of the different genes related to CMT in Brazil

Professor Wilson Marques Jr., from USP Ribeirão Preto (Ribeirão Preto, SP) and collaborators, in 2005, reported the frequency of *PMP22* gene duplication and the clinical findings of individuals in a cohort of 53 families with demyelinating CMT. The 17p11.2-p12 chromosome duplication was present in 42 of 53 (79%) index cases. In those families with *PMP22* duplication, an evident autosomal dominant inheritance was identified in only 8 cases (19%) and 9 cases (22%) were considered sporadic. 25

In 2013, Pasnoor et al., 26 in a collaborative North America and South America epidemiological study on the frequency of several types of neuropathies between the University of Kansas Medical Center, University of Texas Southwestern Medical Center, and Universidade Federal Fluminense, found that CMT accounted for 5.80% of neuropathy cases in the Brazilian center.

**Figure 1:**
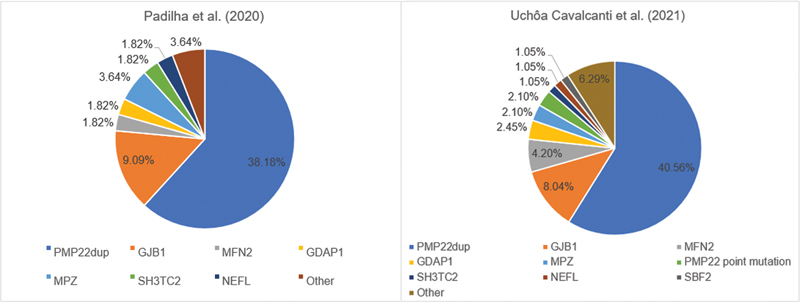
Relative frequencies of CMT-related genes in studies conducted in Brazil.

Padilha et al., 27 in 2020, performed a single-center cross-sectional study at Hospital das Clínicas de Porto Alegre (Porto Alegre, RS) to evaluate the diagnostic yield of a combined molecular diagnostic strategy (MLPA and NGS panel) for 55 CMT index cases. The authors 27 found that *PMP22* and *GJB1* were the most frequent genes related to CMT in the South region of Brazil, representing, respectively, 38.18% and 9.09% of overall CMT cases ( Table 1 and Figure 1 ).

**Table 1:** Relative frequencies of CMT-related genes in studies conducted in Brazil

	Padilha et al. 27	%	Uchôa Cavalcanti et al. 13	%
Index Cases	55	100.00%	286	100.00%
Genetically confirmed	34	61.82%	197	68.88%
Not genetically confirmed	21	38.18%	89	31.12%
**Genes**		%		%
PMP22dup	21	38.18%	116	40.56%
GJB1	5	9.09%	23	8.04%
MFN2	1	1.82%	12	4.20%
GDAP1	1	1.82%	7	2.45%
MPZ	2	3.64%	6	2.10%
PMP22 point mutation	0	0.00%	6	2.10%
SH3TC2	1	1.82%	3	1.05%
NEFL	1	1.82%	3	1.05%
SBF2	0	0.00%	3	1.05%
Other	2	3.64%	18	6.29%

In 2021, Figueiredo et al. 28 reported that the prevalence rate of *GDAP1* among the axonal CMT cases followed at the Neurogenetics Clinic at USP Ribeirão Preto (Ribeirão Preto, SP) was 7.14% (5/70), all of them of recessive inheritance, suggesting that the prevalence was higher than what is observed in most countries. Also, this year, Uchôa Cavalcanti et al. 13 provided further data on the frequency of CMT subtypes and found that *GDAP1* was the fourth more common gene, with a prevalence slightly higher than the *MPZ* , representing 2.45% of the cases in a Brazilian clinical-based population from Rede SARAH de Hospitais de Reabilitação (Brasília, DF) and confirming its importance in our country. Furthermore, this study also corroborated the findings of Padilha et al. 27 on the two most frequent forms of CMT, CMT1A and CMTX1, respectively, and pointed out the presence of rarer forms of CMT, not previously described in our population ( Table 1 and Figure 1 ). 13

### Pain

Pain is a poorly studied phenomenon in CMT, with a frequency between 28.8% and 88% according to previously published works. 29 30 31 32 The study of pain in CMT patients is complex as it is necessary to consider several variables, such as genetic subtype, severity of neuropathy, history of orthopedic injuries (sprains and fractures), presence of musculoskeletal deformities, and overweight/obesity. Sometimes, differentiating pain mechanisms in this condition can be challenging, especially when there is an overlap between them.

In 2012, Ribiere et al. 29 analyzed data from 48 patients with genetically confirmed diagnosis of CMT. Subjects underwent extensive assessment, including evaluation by Visual Analogical Scale (VAS), Hospital Anxiety and Depression Scale (HAD), DN4 scale, and Neuropathic Pain Symptom Inventory (NPSI). They observed that in 66% of cases, patients do report pain, and this is usually moderate, preferentially located in the extremities and in symmetric pattern. In 62.5% of cases, the pain has a neuromuscular origin with a positive DN4 in 50% of cases.

Ramchandren et al., 33 in 2014, reported data collected from 176 children with CMT diagnosis from study sites of the Inherited Neuropathy Consortium, including standardized assessments of pain (Wong-Baker FACES Pain Rating Scale) and standardized clinical assessments and quality-of-life (QOL) outcomes. The authors 33 observed that pain scores do not positively correlate with neuropathy severity but do correlate in limited univariate analyses with measures of ankle inflexibility.

In 2020, Bjelica et al. 31 evaluated 51 CMT1 patients with confirmed molecular diagnosis of PMP22 duplication. They used the International Association for the Study of Pain (IASP) criteria for the diagnosis of neuropathic pain. This type of pain was found in 15 (29.4%) patients with CMT1A, with mean pain intensity of 5.7 ± 2.2 out of 10. It was also observed that the presence of neuropathic pain was associated with worse functional disability and depression. 31

Azevedo et al., 34 in 2021, evaluated 19 patients with a diagnosis of CMT1A. Pain prevalence was 84.2%, with moderate intensity and nociceptive characteristics according to the LANSS scale (75%) and clinical evaluation (50%), but differing from DN4, which found neuropathic pain in most of the patients (56.2%). Mixed pain type was also observed in 43.7% of the patients, according to clinical criteria. The authors 34 also observed that there was a statistically significant correlation between pain intensity and SF-36, suggesting a relation between pain intensity and impact on quality of life.

In 2022, Peretti et al. 32 investigated small fibers involvement and its correlation with pain in different CMT subtypes through a systematic clinical and neurophysiological study, including evaluation by the laser-evoked potentials (LEPs). This study enrolled 50 patients with disease causing-variants in PMP22, MPZ, GJB1, and MFN2. The authors found an overall pain prevalence of 36% and neuropathic pain in 14.6% of patients, with a length-dependent distribution in 85.7% of them. Neuropathic pain prevalence varies among CMT subtypes, being significantly more frequent in CMT1A, and is related to Aδ fibers impairment. 32 This study corroborated the observation of the study of Laurà et al. 30 that neuropathic pain in patients with CMT1A could have its etiology in Aδ fiber dysfunction.

### Pregnancy

Physicians responsible for the follow-up of patients with neuromuscular diseases face several dilemmas related to female fertility, risk to the fetus, ability to perform pregnancy and possible complications during pregnancy and labor, which may eventually require surgical obstetric interventions. Furthermore, many questions have been raised regarding the influence of pregnancies on the natural history of these disorders and data on this issue are scarce. 35 36 37

In the case of CMT, worsening during pregnancy has been previously described. 37 38 39 40 In 1973, Bellina and Deming 38 were the first to publish a case report on a potential association between pregnancy and a possible negative outcome on the natural history of CMT. In 1982, Pollock et al., 39 also published a case report observing worsening of CMT in a previously asymptomatic patient in her third pregnancy and suggested that the pathogenetic mechanisms leading to exacerbation of neuropathy in pregnancy may be related to nerve edema. Brian et al., 41 in 1987, made similar observation in a 20-year-old woman who had progressive weakness of the lower extremities, shortness of breath, and orthopnoea after the fourth gestational month.

Since these brief communications about exacerbation of Charcot-Marie-Tooth disease during pregnancy, that issue in female patients with CMT has become a matter of concern for health teams. Although CMT apparently does not affect women's ability to carry out a pregnancy in most case series, there are conflicting data regarding the risk of complications related to childbirth and changes in neurological condition during gestation. 37 40 42 43 44 45 46

In 1993, Rudnik-Schöneborn et al. 47 evaluated the courses and outcomes of 45 pregnancies in 21 patients with CMT1, which were investigated through postal questionnaires. Eight out of 21 patients (38.09%) reported worsening in 21/45 gestations (46.66%). The main complaint after the 1 ^st^ trimester was increasing weakness in the lower extremities (17/21). Patients who had pregnancy-associated progression in the first gestation (7/21) experienced similar deterioration in subsequent pregnancies (10/11). 47

Hoff et al., 36 in 2005, reported data from the Medical Birth Registry of Norway. One-hundred and eight births by mothers with CMT were identified. The authors reported that women with CMT had a higher occurrence of presentation anomalies and postpartum bleeding. The rate of operative delivery was twice that of the reference group, and forceps was used three times as often in the CMT group. The majority of CMT caesarean sections were emergency sections. 36

In 2016, Leal, 43 in her doctoral thesis, evaluated the influence of pregnancy on 51 female patients with CMT1A. One-hundred and thirty pregnancies were evaluated. Twenty-nine of 51 women (56%) reported worsening of their neurological status during 62/130 (47.69%) pregnancies: 8/29 (27.58%) patients developed positive sensory symptoms, 6/29 (20.69%) women reported more frequent falls, 3/29 (10.34%) mothers reported progressive weakness, 2/29 (6.90%) patients described symptoms of a sensitive ataxia, 23/29 (79.31%) women had painful cramps. Two women were restricted to a wheelchair in the 3 ^rd^ trimester and until 3 months after delivery. One of them had severe sensitive ataxia and the other had worsening of weakness in four limbs. The deterioration was temporary in 17/29 (58.62%) gestations. 43

Rudnik-Schöneborn et al., 42 in 2020, conducted a clinical cohort study and cross-sectional study within the German CMT-NET, involving 54 women and 98 pregnancies. There was no increased newborn morbidity and mortality. Approximately one-third of patients reported exacerbation of CMT disease during or after pregnancy. No adverse effects of anesthesia were reported. 42

In the same year, Pisciotta et al. 40 published the results of their study involving data on 193 pregnancies from 86 women with CMT, with 157 deliveries (81.4%) after a mean of 38.6 gestational weeks. They observed higher rates of placenta previa, abnormal presentations, and preterm deliveries in CMT, but pregnancy outcome and newborn weight and health were like those of the reference populations. Worsening of CMT is not infrequent and occurs not only in CMT1A. 40

There is concern about the methods of some studies, often performed retrospectively, restricted to specific subtypes of CMT, and without analyzing more objective parameters in relation to the neurological condition (use of standardized scores and measures). It is also noteworthy that obstetric care varies in terms of quality in most of the populations studied (prepregnancy counselling, number of prenatal consultations, accessibility to referral centers). In our country, there is still a bias toward the indication of caesarean section, with more than 50% of deliveries performed by this route, often not motivated by obstetric reasons. 48

Another crucial point were the advances in preimplantation genetic diagnosis (PGD) for numerical and structural chromosomal anomalies and monogenic disorders. Preimplantation genetic diagnosis has been successfully used to differentiate affected and unaffected embryos before transfer in human in vitro fertilization programs, allowing to avoid the onset of affected pregnancies. 49 In 1998, De Vos et al. 50 reported the use of polymerase chain reaction (PCR)-based assay developed for the detection of duplication of 1.5 Mb on chromosome 17 for PGD in a couple whose male individual had confirmed molecular diagnosis of CMT1A, resulting in an unaffected singleton pregnancy. Over the years, other case reports were described in CMT patients, corroborating that this technique proved to be a viable option during reproductive counselling for these individuals. 50 51 52

### Respiratory features

Although prominent respiratory dysfunction is not a frequent feature of patients with CMT, it can occur and is probably a neglected aspect. There are several case reports with descriptions of vocal cord and diaphragmatic paresis, including the need for noninvasive ventilation. 53 54 55 56 Special attention should be given to patients with severe forms, restricted to wheelchair, and in those with significant scoliosis.

In 2015, De Carvalho Alcântara et al. 57 analyzed pulmonary function test, polysomnography, diaphragm ultrasound and phrenic nerve CMAP of 16 individuals (6 male and 10 females) from 8 families with CMT1A. Maximal inspiratory pressure (MIP) was reduced in 5 (31.25%) and maximal expiratory pressure (MEP) in 12 patients (75%). Restrictive respiratory dysfunction on spirometry was detected only in the most disabled patient. A significant increase in the sleep apnea and phrenic nerve index was observed on ultrasound in patients with CMT1A. The authors 57 suggested that axonal degeneration of nerves directed to respiratory muscles could explain the increased prevalence of weakness in respiratory muscles in individuals with CMT1A.

Spiesshoefer et al., 58 in 2019, evaluated the phrenic nerve conduction and the inspiratory and expiratory function of 19 adults with CMT1A, 13 females and 6 males. The authors found that forced vital capacity (FVC), MIP, MEP, and peak cough flow were significantly lower in CMT1A patients than in controls. They 58 also observed that diaphragm motor evoked potentials and compound muscle action potentials were delayed in CMT1A patients, and that diaphragmatic dysfunction was related to disease severity, as measured by CMT-NSv2, being present even in patients who are not wheelchair-bound.

Uchôa Cavalcanti et al., 13 in 2021, found that changes in respiratory function were frequent in patients with recessive forms of CMT. In 7 of 8 patients who had undergone a pulmonary function test, a restrictive pattern was detected, characterized by the presence of reduced FVC and normal FEV1/FVC ratio. The authors also observed vocal cord paralysis in one patient with the GDAP1 likely pathogenic variant c.355C > A (p.Pro119Thr) in homozygous state and in one patient with the homozygous novel variant c.943 + 2 T > C in the NDGR1. 13

### Treatment

In the last decades, with important advances in the understanding of diverse molecular mechanism involved in CMT pathogenesis and in the field of genetic therapies, including RNA interference and gene editing techniques, the first steps were taken toward research into potential treatments for hereditary neuropathies. 7 15 59 60 61

Many treatment approaches, including gene silencing or replacement therapies, and small molecule treatments are currently in preclinical phase. 15 Some of these potential therapies approach are disease-specific targeted, while others address common pathways shared by many CMT subtypes ( Table 2 ). As those promising treatments have the possibility of clinical translation, better outcome measures, novel biomarkers and appropriate trial designs are essential to the validation of novel treatments for CMT patients.

**Table 2:** Recent studies on possible treatments for Charcot-Marie-Tooth disease

Study	Year	Type	Authors
Coenzyme Q10 therapy in hereditary motor sensory neuropathy type VI with novel mitofusin 2 mutation	2012	Case report	Takahashi et al. 78
Polytherapy with a combination of three repurposed drugs (PXT3003) down-regulates Pmp22 overexpression and improves myelination, axonal and functional parameters in models of CMT1A neuropathy	2014	Preclinical	Chumakov et al. 62
PMP22 antisense oligonucleotides reverse Charcot-Marie-Tooth disease type 1A features in rodent models	2017	Preclinical	Zhao et al. 68
MFN2 agonists reverse mitochondrial defects in preclinical models of Charcot-Marie-Tooth disease type 2A	2018	Preclinical	Rocha et al. 80
Early short-term PXT3003 combinational therapy delays disease onset in a transgenic rat model of Charcot-Marie-Tooth disease 1A (CMT1A)	2019	Preclinical	Prukop et al. 63
Gene replacement therapy after neuropathy onset provides therapeutic benefit in a model of CMT1X.	2019	Preclinical	Kagiava et al. 76
Curcumin–cyclodextrin/cellulose nanocrystals improve the phenotype of Charcot-Marie-Tooth-1A transgenic rats through the reduction of oxidative stress	2020	Preclinical	Caillaud et al. 70
Targeted PMP22 TATA-box editing by CRISPR/Cas9 reduces demyelinating neuropathy of Charcot-Marie-Tooth disease type 1A in mice	2020	Preclinical	Lee et al. 69
AAV2/9-mediated silencing of PMP22 prevents the development of pathological features in a rat model of Charcot-Marie-Tooth disease 1 A	2021	Preclinical	Gautier et al. 71
A double-blind, placebo-controlled, randomized trial of PXT3003 for the treatment of Charcot-Marie-Tooth type 1A	2021	Phase III	Attarian et al. 65
Treatment with IFB-088 improves neuropathy in CMT1A and CMT1B mice	2022	Preclinical	Bai et al. 72

#### PMP22

In 2014, Chumakov et al. 62 described the effects of PTX3003, a combination of already marketed and known medications (baclofen, sorbitol, and naltrexone), on PMP22 transgenic CMT1A rats, with improved myelination of small fibers and increased nerve conduction. They 62 also demonstrated that PXT3003 was able to downregulate the expression of PMP22 in cultured Schwannoma cells more efficiently than single drugs. Later, in 2019, Prukop et al. 63 reported an early postnatal, short-term treatment with PXT3003 in CMT1A rats that delayed disease onset into adulthood. The authors also observed that PXT3003 reduced PMP22 mRNA overexpression and improved the misbalanced downstream PI3K-AKT / MEK-ERK signaling pathway. 63 In 2014, a phase II trial confirmed the safety and tolerability of PXT3003 in CMT1A patients. 64 Attarian et al., 65 in 2021, in a phase III trial, randomly assigned 323 subjects with mild-to-moderate CMT1A to receive high- or low-dose of PXT3003 or placebo. Both PXT3003 doses were safe and well-tolerated and high-dose PXT3003 demonstrated significant improvement in the Overall Neuropathy Limitations Scale total score when compared with placebo. 65

Also, in 2018, Fledrich et al. 66 showed that myelinating Schwann cells in a rat model of CMT1A exhibit a developmental defect that includes reduced transcription of genes required for myelin lipid biosynthesis. Further studies about this altered lipid biosynthesis pathways were conducted by Visigalli et al. 67 in 2020. The authors 67 demonstrated a comprehensive lipid profiling in experimental and human CMT1A, disclosing a previously unknown specific alteration of sphingolipid and glycerophospholipid metabolism. They 67 also observed that sphingolipid and glycerophospholipid were not merely reduced, but additionally their expression was aberrant, contributing to the ultrastructural abnormalities in the internode myelin.

In 2018, Zhao et al. 68 demonstrated that antisense oligonucleotides effectively suppress PMP22 mRNA in affected nerves in two murine CMT1A models. They 68 also reported that reduction of PMP22 mRNA in skin biopsies from ASO-treated rats were a suitable biomarker for evaluating response to therapy.

Lee et al., 69 in 2020, targeted TATA-box of human PMP22 promoter to normalize overexpressed PMP22 level in a mouse model of CMT1A harboring multiple copies of human PMP22. The authors 69 demonstrated that intraneural delivery of CRISPR/Cas9 designed to target TATA-box of PMP22 before the onset of disease downregulates gene expression of PMP22 and preserves both myelin and axons.

Caillaud et al., 70 in 2020, investigated the therapeutic potential of cyclodextrin/cellulose nanocrystals of curcumin (Nano-Cur) in vitro in Schwann cells and in vivo in the transgenic CMT1A rat model. The authors 70 observed that Nano-Cur reduced reactive oxygen species and improved mitochondrial membrane potential in Schwann cells. In vivo experiments showed that intraperitoneal injection of Nano-Cur treatment strongly enhanced the bioavailability of curcumin and significantly improved sensory-motor functions (grip strength, balance performance, and mechanical and thermal sensitivities). Sensory and motor nerve conduction velocities were also improved, and histological and biochemical analyses demonstrated that myelin sheath thickness and myelin proteins expression were increased. 70

In 2021, Gautier et al. 71 evaluated the safety and efficacy of recombinant adeno-associated viral vector targeting PMP22 mRNA in animal models of CMT1A. The treatment restores expression levels of PMP22 comparable to wild-type conditions and increased myelination and prevented motor and sensory impairments over a 12-month period in a rat model of CMT1A. 71

Bai et al., 72 in 2022, administered Sephin1/IFB-088/icerguestat, an unfolded protein response modulator, to heterozygous MPZ R98C and C3-PMP22 mice. The authors 72 analyzed behavioral, neurophysiological, morphological, and biochemical measures of mice and observed improvement in motor function and neurophysiological parameters. Myelination, demonstrated by g-ratios and myelin thickness, improved in CMT1B and CMT1A mice and markers of unfolded protein response activation returned to wild-type values. 72

#### GJB1

Mones et al., 73 in 2015, observed that transgenic cells from mice model exhibit CamKII over-stimulation, a phenomenon that has been linked to mitotic instability. They also demonstrate that connexon activity is partially restored with CamKII inhibitors. They 73 reported that fibroblasts from CMTX1 patients present a phenotype similar to transgenic lines that can be corrected by CamKII inhibitors. These findings presented a possible path for the development of therapeutic strategies for the treatment of patients with CMTX1.

CMTX1 disease is usually caused by loss-of-function variants and therefore this data supports the concept that gene replacement therapy can be potentially beneficial. 74 Kagiava et al. 75 76 demonstrated that intrathecal gene therapy in GJB1 null mice models altered the peripheral nervous system pathology, improving the functional and morphological properties of the demyelinating neuropathy. The authors 75 76 also observed that plasma neurofilament light levels were also ameliorated in fully treated mice. Future studies will be needed to assess the viability of this approach in clinical trials.

#### MFN2

MFN2 mediates the fusion of mitochondria and contributes to the dynamic balance between fusion and fission which determines mitochondria morphology. It iso also involved in other aspects of mitochondrial metabolism, as well as cell signaling and apoptosis. 77

Takahashi et al., 78 in 2012, reported the case of a 37-year-old patient with hereditary sensory motor neuropathy and optic atrophy with the variant c.1091G > A (p.Arg364Gln) in the MFN2. The patient was treated with high dose of Coenzyme Q10 (CoQ10) (200 mg/day) for 8 months. The authors 78 observed partial improvement in visual impairment after therapy and hypothesized that high dose of CoQ10 may improve the prognosis of subacute visual impairment in HSMN VI. In 2015, Mourier et al. 79 found a novel role for MFN2 in the maintenance of the terpenoid biosynthesis pathway, which is necessary for mitochondrial coenzyme Q biosynthesis. They 79 also observed that reduced respiratory chain function in cells lacking MFN2 can be partially rescued by coenzyme Q10 supplementation, which could suggest a possible therapeutic strategy for patients with disease-causing variants in the *MFN2* gene.

Another promising therapeutic target for CMT2A involves mitofusin agonists, which have been shown to normalize mitochondrial trafficking within sciatic nerves of MFN2 Thr105Met mice. 80

In conclusion, there are still many challenges regarding CMT care. Many individuals still do not have a confirmatory molecular diagnosis, either due to limitations of current investigation methods or due to difficulty in accessing centers for genetic testing. We also know that there are many doubts about the reasons that lead to intra- and interfamilial variability in some forms of CMT, where some individuals with the same variant are more severely affected than others.

For most patients living in Brazil, there still is difficulty in accessing reference centers for care and rehabilitation. Pain appears to be a neglected symptom and part of a multifactorial context. Although rehabilitation plays an essential role in maintaining the quality of life of patients with CMT, data on what would be the best approaches are insufficient in the literature and more scientific studies are needed in this area.

Even today, many patients do not receive genetic counselling properly and there are still doubts about the potential risks related to pregnancy. Understanding the risks and planning appropriately are very important for women with CMT considering pregnancy and their health care providers, especially because of the options that advances in the area of preimplantation diagnosis have brought in recent years.

In view of the new treatment perspectives, it is important that we know better the frequency of the different genetic subtypes in our population and that patients have the opportunity to be referred to centers that can meet the increasing complexity in the care of these individuals.
